# TLR4 Agonist and Hypoxia Synergistically Promote the Formation of TLR4/NF-*κ*B/HIF-1*α* Loop in Human Epithelial Ovarian Cancer

**DOI:** 10.1155/2022/4201262

**Published:** 2022-04-14

**Authors:** Bin Zhao, Xiulong Niu, Suhui Huang, Jing Yang, Yiyi Wei, Xiujuan Wang, Junhong Wang, Yue Wang, Xiaoqin Guo

**Affiliations:** ^1^School of Integrative Medicine, Tianjin University of Traditional Chinese Medicine, Tianjin 301617, China; ^2^Department of Neurology, Tianjin Medical University General Hospital, Tianjin 300052, China; ^3^Department of Prevention & Therapy of Skin Disease in the Security Environment, Characteristic Medical Center of Chinese People's Armed Police Force, Tianjin 300162, China; ^4^Department of Pathogenic Biology and Immunology, Logistics University of Chinese People's Armed Police Forces, Tianjin 300309, China; ^5^Department of Disease Control and Prevention, Tibetan Armed Police Force Hospital, Lhasa, Tibet 850000, China; ^6^Institute of Prevention and Treatment of Cardiovascular Diseases in Alpine Environment of Plateau, Characteristic Medical Center of Chinese People's Armed Police Force, Tianjin 300162, China; ^7^Institute of Disaster and Emergency Medicine, Tianjin University, Tianjin 300072, China

## Abstract

Inflammation and hypoxia are involved in numerous cancer progressions. Reportedly, the toll-like receptor 4 (TLR4)/nuclear factor kappa B (NF-*κ*B) pathway and hypoxia-inducible factor-1*α* (HIF-1*α*) are activated and closely related to the chemoresistance and poor prognosis of epithelial ovarian cancer (EOC). However, the potential correlation between TLR4/NF-*κ*B and HIF-1*α* remains largely unknown in EOC. In our study, the possible positive correlation among TLR4, NF-*κ*B, and HIF-1*α* proteins was investigated in the EOC tissues. Our in vitro results demonstrated that LPS can induce and activate HIF-1*α* through the TLR4/NF-*κ*B signaling in A2780 and SKOV3 cells. Moreover, hypoxia-induced TLR4 expression and the downstream transcriptional activity of NF-*κ*B were HIF-1*α*-dependent. The cross talk between the TLR4/NF-*κ*B signaling pathway and HIF-1*α* was also confirmed in the nude mice xenograft model. Therefore, we first proposed the formation of a TLR4/NF-*κ*B/HIF-1*α* loop in EOC. The positive feedback loop enhanced the susceptibility and responsiveness to inflammation and hypoxia, which synergistically promote the initiation and progression of EOC. The novel mechanism may act as a future therapeutic candidate for the treatment of EOC.

## 1. Introduction

Epithelial ovarian cancer (EOC) is a leading lethal cancer among the gynecological malignancies occurring worldwide [1]. In fact, 70–75% of EOC patients are detected at advanced stages, which converts into a high mortality rate. Although the popular treatment options currently available include surgery and chemotherapy, they are restricted by cases of frequent recurrence, and the five-year survival rate is approximately 30% [2, 3]. Therefore, it is necessary to further elucidate the molecular mechanisms of ovarian carcinogenesis and to improve the treatment modalities for EOC.

Chronic inflammation and hypoxia are linked to the tumor microenvironment [4]. Inflammation is a crucial risk stressor that is associated with tumor survival and invasion. Toll-like receptor 4 (TLR4), a member of the TLRs family, which is the indispensable receptor for lipopolysaccharide (LPS), is detected in a variety of tumors, including in ovarian malignant tumors [5, 6]. TLR4 upon recognition of its ligand LPS initiates downstream inflammatory responses. Past studies have demonstrated that the activation of TLR4 signaling generates proinflammatory cytokines and antiapoptotic proteins, which in turn contributes to the growth, metastasis, and chemoresistance in ovarian cancer [7, 8]. In response to LPS, TLR4 mediates both myeloid differentiation factor 88- (MyD88-) dependent and MyD88-independent signaling pathways. The MyD88-dependent pathway leads to the expression of proinflammatory factors by activating the early phase of NF-*κ*B. The MyD88-independent signaling pathway induces the late-phase activation of NF-*κ*B, which triggers the expression of interferon-beta [9]. NF-*κ*B, a critical regulator of inflammation, is believed to be a key link between inflammation and tumor [10]. Therefore, NF-*κ*B has become an excellent target for the treatment of human tumors [11].

In addition to inflammation, hypoxia within tumors has also been shown to contribute to tumor progression. Hypoxia is an essential feature existing in most solid tumors [12]. Hypoxia-inducible factor-1 (HIF-1), which is a key transcription factor of hypoxia response, is a heterodimer that comprises of an inducible subunit, HIF-1*α*, and a constitutive subunit, HIF-1*β*. Under hypoxia, HIF-1*α* accumulates, forms a dimer with HIF-1*β*, and modulates more than 100 hypoxia-related genes [13].

Past studies have demonstrated that the TLR4/NF-*κ*B pathway can stabilize and accumulate HIF-1*α* under normoxic conditions. In macrophages and dendritic cells, LPS can mediate HIF-1*α* expression by activating NF-*κ*B [14, 15]. More recently, peroxiredoxin 1 (Prx1), a TLR4 endogenous ligand, has also been identified to increase the interaction between NF-*κ*B and HIF-1*α* promoters, which contributes to the enhancement of the HIF-1*α* mRNA levels along with augmentation of the HIF-1 activity [16]. Therefore, HIF-1*α* is a key link between hypoxia and inflammation [17]. Kim and his colleagues documented that the expression level of TLR4 was upregulated through HIF-1*α* in macrophages under hypoxia. Furthermore, the PI3K/Akt pathway, which promotes the translocation and activation of HIF-1, contributes to hypoxia-induced TLR4 expression [18, 19]. These factors suggest that the cross talk between the TLR4/NF-*κ*B signaling pathway and HIF-1*α* may form a positive feedback loop that promotes the susceptibility to inflammatory signals under hypoxia. Reportedly, the interaction between the TLR4/NF-*κ*B signaling pathway and HIF-1*α* contributes to tissue damage and tumor progression in pancreatic ductal adenocarcinoma, oral squamous cell carcinoma, and lung ischemia-reperfusion injury [20–22]. However, the presence of any association between TLR4 and HIF-1*α* signaling in EOC remains unclear.

In the present study, we detected a positive association among TLR4, NF-*κ*B, and HIF-1*α* protein levels in the EOC tissues. Further studies demonstrated the formation of TLR4/NF-*κ*B/HIF-1*α* positive feed-forward loop in EOC cells. These findings are closely associated with inflammation and hypoxia, which synergistically promote the development of ovarian cancer. We believe that our study will contribute to developing novel targeted therapies for EOC treatment.

## 2. Materials and Methods

### 2.1. Materials

Solutions and chemicals listed in the study are as follows: lipopolysaccharides (LPS) extracted from *Escherichia coli* O55:B5, cobalt chloride (CoCl_2_), pyrrolidine-dithiocarbamic acid, and ammonium salt (PDTC) (Sigma-Aldrich Inc., Germany); 6R-[[(2-Chloro-4-fluorophenyl)amino]sulfonyl]-1-cyclohexene-1-carboxylic acid and ester (TAK-242); and 5-[1-(phenylmethyl)-1H-indazol-3-yl]-2-furanmethanol (YC-1) (Cayman Chemical Inc., USA); anti-TLR4 antibody (1 : 500 dilution, cat. no.35577; Signaling Antibody, Inc., USA); anti-MyD88 antibody (1 : 1000 dilution, cat. no. ab133739; Abcam, Cambridge, UK); anti-NF-*κ*Bp65 (1 : 1000 dilution, cat. no.8242), anti-phospher-NF-*κ*Bp65 (Ser536) (1 : 1000 dilution, cat. no.3033), and anti-HIF-1*α* antibodies (1 : 1000 dilution,; cat. no.14179) (Cell Signaling Technology Inc., USA); anti-*β*-actin antibody (1 : 8000 dilution,; cat. no.RM2001), goat-anti-mouse secondary antibody (1 : 8000 dilution,; cat. no.RM3001), and goat-anti-rabbit secondary antibody (1 : 8000 dilution,; cat. no.RM3002) (Beijing Ray Antibody Biotech Company, Beijing, China); RPMI 1640 and DMEM-H medium (Invitrogen Co., USA); and fetal bovine serum (FBS; LONSA SCIENCE SRL., USA). All chemicals were of 99% purity.

### 2.2. Cell Culture

Human epithelial ovarian cancer cell lines A2780, ES-2, SKOV3, and OVCAR3 were sourced from ATCC. Four cell lines were maintained in RPMI 1640 with 10% FBS or DMEM-H with 10% FBS, respectively. To establish a model of cellular hypoxia, we cultured the ovarian cancer cell lines in 1% oxygen (hypoxia group) instead of 21% (normoxia or control group). CoCl_2_ dissolving in 1% FBS medium was used to simulate chemical hypoxia when incubated with cells, and others in cell experiments, like PDTC, were utilized similarly to cobalt chloride. The nonpolar reagents such as TAK-242 and YC-1 were dissolved in a DMSO medium to a concentrated liquid, followed by dilution to different concentrations with 1% FBS medium. In addition, the concentrations of the reagents mentioned in this paper were determined through preliminary tests.

### 2.3. Immunohistochemistry Analysis

The levels of TLR4, NF-*κ*Bp65, and HIF-1*α* in human ovarian normal tissues (*n* = 10), human benign ovarian tumors (*n* = 10), human borderline ovarian tumors (*n* = 10), and well-differentiated (*n* = 12) and poorly differentiated human EOC tissues (*n* = 14) were assessed by immunohistochemistry analysis. The tissue specimens were obtained from the Department of Pathology, blinded for peer review, fixed in 10% formalin buffer, and embedded in paraffin. The sections (5 *μ*m thick) were prepared and subjected to testing by using the SP Kit (KeyGen, Nanjing, China) as per the manufacturer's instruction, followed by overnight incubation with anti-TLR4, anti-NF-*κ*Bp65, and anti-HIF-1*α* (1 : 200) antibodies at 4°C in a wet box. After washing thrice with PBS, the biotin-labeled secondary antibody working solution was added drop-wise and incubated at 37°C for 20 min. Next, the sample was incubated with horseradish peroxidase-labeled streptavidin at 37°C for 30 min (washed thrice with PBS). The DAB coloring solution was added to the tissue section for color development. After the optimal coloring time was reached based on the observation under a microscope, the section was washed under running water for 2 min to terminate any further color development. The value of integrated option density (IOD) was evaluated by the Image-Pro Plus 6.0 software (Media Cybernetics, Silver Spring, MD, USA), represented as the summational IODs of 5 random scales of each paraffin section. The Ethics Committee approved the study.

### 2.4. Real-Time Fluorescence Quantitative PCR

We used TRIzol (Invitrogen, Carlsbad, CA, USA) to extract the total RNA in the cells according to the manufacturer's protocol and then detected mRNA by *TransStart®* Top Green qPCR SuperMix (TransGen Biotech, China). Real-time fluorescence quantitative PCR (qRT-PCR) was executed by CFX96 Connect Real-Time PCR Systems (Bio-Rad, USA). The cycling program was as follows: initial denaturation step at 95°C for 3 min, followed by denaturation at 95°C for 10 s, annealing at 60°C for 15 s, and extension at 72°C for 10 s for 40 cycles. All qRT-PCR reactions were performed in triplicate, and the test gene was normalized to the *β*-actin. The relative expression of the gene was evaluated based on the 2^−*ΔΔ*Ct^ method. The primer sequences used in the experiment were as follows: TLR4 (203 bp), (F)5′-GATAGCGAGCCACGCATTCA-3′ and (R)5′-AAGCTCTGGGTTTCATGCCA-3′; MyD88 (170 bp), (F)5′- TGGCACCTGTGTCTGGTCTA-3′ and (R)5′-ACTTGATGGGGATCAGTCGC-3′; NF-*κ*Bp65 (240 bp), (F)5′-GCGAGAGGAGCACAGATACC-3′ and (R)5′-AGGGGTTGTTGTTGGTCTGG-3′; HIF-1*α* (290 bp), (F)5′-ACAAGCCACCTGAGGAGAGG-3′ and (R)5′-TGGCTGCATCTCGAGACTTT-3′; and *β*-actin (150 bp), (F)5′-GCACTCTTCCAGCCTTCCTT-3′ and (R)5′-AATGCCAGGGTACATGGTGG-3′.

### 2.5. Western Blotting

We lysed cells with lysis buffer (KeyGen, Nanjing, China) on an ice bath, then centrifuged the resultant at 12000 rpm at 4°C for 30 min, and then used the BCA kit (Pierce Biochemicals, USA) to detect the protein concentration. 30–80 *μ*g proteins were separated on 10-12% SDS-PAGE and then transferred onto a polyvinylidene fluoride membrane. After blocking the antigen, the membrane was incubated overnight at 4°C with primary antibodies and then washed with TBST and incubated with secondary antibodies for 1 h. We detected the target protein levels using enhanced chemiluminescence reagents (Millipore, USA). The values of the target protein were normalized to the corresponding *β*-actin values.

### 2.6. Cell Viability Assays

The MTT assay was performed to detect the cell viability following hypoxia. Briefly, A2780 or SKOV3 cells (4 × 10^3^ cells per well) were inoculated into 96-well plates overnight, followed by replacement with a medium with 1% FBS, and then cultured for 24 h. The resultant cells were treated with 1% O_2_ or 50, 100, 150, 200, and 300 *μ*M CoCl_2_, respectively. After 24 h, the cells were incubated with 0.5 mg/mL MTT solution (PBS) for 4 h. After dissolving with acid-isopropanol, the plates were detected at a wavelength of 490 nm.

### 2.7. Plasmids

The kind gifts of the pCMVh-HA-ssHIF-1*α* and pCMVh-HA plasmids were obtained from Dr. Andrew L. Kung (Dana-Farber Cancer Institute, USA). HIF-1*α* shRNA sequences were obtained from Suzhou Genepharma Co., Ltd. (homo 1614; Suzhou, China). To construct the pGPU6-HIF-1*α* shRNA plasmid, the HIF-1*α* shRNA sequences were synthesized, pair annealed, and subcloned into the eukaryotic expression vector pGPU6. The recombinant vectors were transiently transfected into A2780 and SKOV3 cells by Lipofectamine™ 2000 (Invitrogen, Carlsbad, CA, USA). pGL3-HRE6-luc and pGL3-NF-*κ*B-Luc reporter plasmids, a 516 bp fragment harboring 6 copies of the hypoxia-response element (HRE) sequences or NF-*κ*B sequences, were amplified from HEC-1-B genomic DNA and cloned into the pGL3-basic vector as per the manufacturer's instructions.

### 2.8. Transient Transfection

The HIF-1*α* knockdown or overexpression and the luciferase-expressing cells were established by transfecting with the Lipofectamine™ 2000. Cells were plated into 6-well plates until a confluence of 90-95% was reached. The cells were transiently transfected with 4 *μ*g of DNA containing the target gene fragment, for instance, ssHIF-1*α*, HIF-1*α* shRN, HRE6-luc, and NF-*κ*B-Luc reporter, among others.

### 2.9. Immunofluorescence Assay

Cells cultured on laser focusing dishes were fixed in 4% paraformaldehyde and permeabilized in 0.5% Triton X-100. Fixed cells were incubated in blocking buffer and then with anti-NF-*κ*Bp65 (FITC-conjugated). Finally, the dishes were incubated with DAPI in PBS and observed with a fluorescence confocal microscope (TSC Sp8, Leica, Germany).

### 2.10. Luciferase Assays

The luciferase activities were detected by reagents purchased from Promega (Madison, WI, USA). The activity of *β*-galactosidase was measured using the *β*-galactosidase Enzyme Assay System (Sigma, St. Louis, MO, USA). The relative luciferase activities were normalized to the corresponding *β*-galactosidase activities.

### 2.11. Xenograft Mouse Model

4-week-old female BALB/c nude mice (weight: 18-22 g, *n* = 32) were obtained from the Institute of Materia Medica, blinded per author guidelines, and bred under the SPF condition. After 1 week, a total of 5 × 10^6^ SKOV3 cells were collected, washed thrice with saline, and subcutaneously implanted into the right-sided flanks of the nude mice. At 7 days of implantation, the tumor became observable. Tumor dimensions were measured every alternate day. A total of 32 mice showed a tumor diameter of approximately 9 mm at 21 days after implantation and were randomly divided into 8 groups. Using the intratumoral injection technique, TAK-242, PDTC, or YC-1 or their common vector DMSO/saline was injected into the xenograft tumors, and LPS or saline was injected 1 h later in the same inlet. After 24 h, mice were sacrificed, and the tumors were excised, weighed, and snap-frozen for RNA and protein extractions or paraffin-embedded for H&E dye (Solarbio, Science and Technology Co., Ltd.). The animal procedure was approved by the Ethics Committee.

### 2.12. Statistical Analyses

Data were analyzed with the IBM SPSS21.0 software package (SPSS, Statistical Product and Service Solution Chicago). Differences of multiple sets of measurement data were performed using one-way or two-way ANOVA. The LSD *t* test was used for the post hoc analysis when the variances were equal, and Dunnett's T3 test was used for unequal variances. Univariate association among clinical samples was assessed by the Chi-square test. *P* < 0.05 was set as statistically significant.

## 3. Results

### 3.1. Positive Correlation Existed between TLR4 Level and the Progression of EOC, and the Levels of NF-*κ*Bp65 and HIF-1*α* in Clinical Specimens

**Figure 1:**
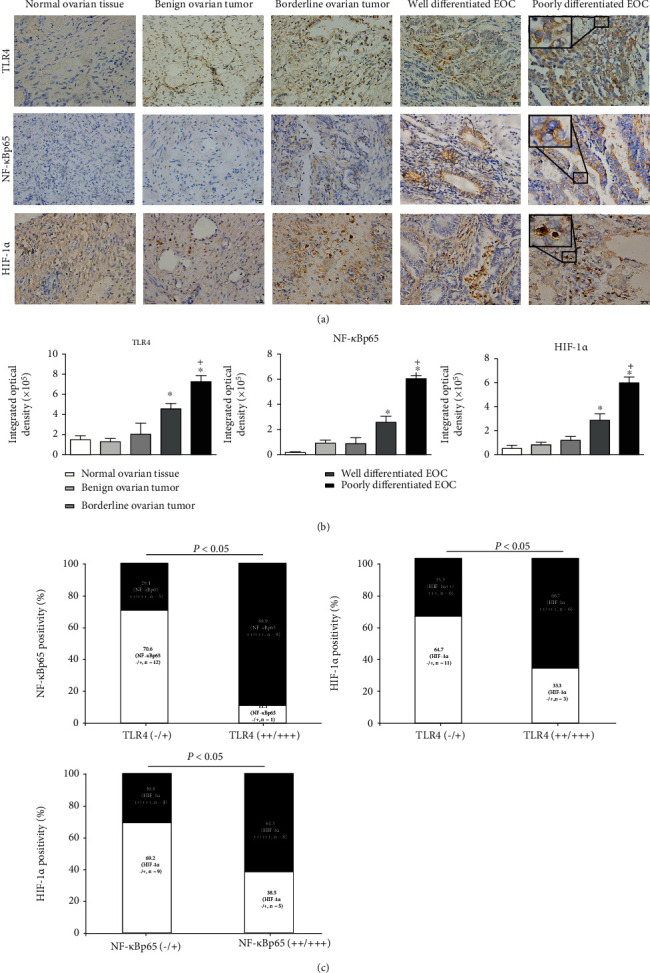
Expression of TLR4, NF-*κ*Bp65, and HIF-1*α* in clinical settings. (a) Representative images by IHC staining for TLR4, NF-*κ*Bp65, and HIF-1*α* in normal ovary tissues, benign or borderline ovarian tumors, and epithelial ovarian cancers. Magnification ×200, inset ×600. (b) The integrated optical density (IOD) values of TLR4, NF-*κ*Bp65, and HIF-1*α* in normal ovary tissues, benign or borderline ovarian tumors, and epithelial ovarian cancers. Data are presented as mean ± SD. ^∗^*P* < 0.05 vs. the normal ovary tissues group, ^+^*P* < 0.05 vs. the well-differentiated EOC group. (c) Percent of NF-*κ*Bp65 positive cells (NF-*κ*Bp65^+ve^ cells) in EOC specimens grouped based on low (-/+) or high (++/+++) TLR4 level and HIF-1*α*^+ve^ cells grouped on the basis of TLR4 level as well as HIF-1*α*^+ve^ cells grouped on the basis of the NF-*κ*Bp65 level. The number of cases analyzed is shown, and the intensity of IHC staining on each section was evaluated independently by two pathologists using the 4-step grading system via IOD analysis, that is, “-, +, ++, and +++” for “negative, low, high, and extremely high,” respectively (-: IODs ≤ 1.0 × 10^5^; +: 1.0 × 10^5^ < IODs ≤ 3.0 × 10^5^; ++: 3.0 × 10^5^ < IODs ≤ 5.0 × 10^5^; and +++: IODs > 5.0 × 10^5^).

To confirm the presence of a correlation among TLR4, NF-*κ*Bp65, and HIF-1*α*, we first determined the status of the abovementioned proteins in human normal ovary tissues and the clinical specimens of EOC by immunohistochemistry (IHC). The representative images (inset in Figure 1(a)) illustrated that TLR4 was mainly expressed on the cell surface and cytoplasm, while NF-*κ*Bp65 mainly existed in the cytoplasm and HIF-1*α* existed in the cytoplasm or nucleus. The normal ovary tissues, benign ovarian tumors, and borderline ovarian tumors were studied to reveal that TLR4, NF-*κ*Bp65, and HIF-1*α* were expressed at a low level, while as cancer progressed, the proportion of the TLR4^+ve^, NF-*κ*Bp65^+ve^, and HIF-1*α*^+ve^ EOC cells were significantly increased (Figure 1(a)). As illustrated in Figure 1(b), the integrated optical density (IOD) value analysis of positively stained settings indicated that the increased levels of TLR4, NF-*κ*Bp65, and HIF-1*α* were observed in the well- or poorly differentiated EOC specimens in comparison with the normal ovarian tissues (all *P* < 0.05). Meanwhile, compared with the well-differentiated EOC, the IOD values of TLR4^+ve^, NF-*κ*Bp65^+ve^, and HIF-1*α*^+ve^ cells obtained from poorly differentiated EOC were significantly higher (all *P* < 0.05). More importantly, further analysis revealed that, in the clinical specimens of EOC, the TLR4 level was significantly positively related to the levels of NF-*κ*Bp65 and HIF-1*α*, respectively (Figure 1(c)). Among the TLR4 strongly positive specimens (++/+++, IODs > 3.0 × 10^5^), the percentage of low NF-*κ*Bp65 or HIF-1*α* expression (-/+, IODs ≤ 3.0 × 10^5^) was only approximately 11.1% or 33.3%, whereas high NF-*κ*Bp65 or HIF-1*α* expression was approximately 88.9% or 66.7%. In addition, we also found that a positive correlation existed with the NF-*κ*Bp65 levels and HIF-1*α*. Based on the IHC figures in Figure 1(a) and the IOD values in Figure 1(b), we calculated the percentage of NF-*κ*B p65^+ve^ cells, as well as HIF-1*α*^+ve^ cells in specimens of low (-/+) or high (++/+++) TLR4 levels (Figure 1(c)). Figure 1(c) results show the possibility of a positive correlation among TLR4, NF-*κ*B, and HIF-1*α* expression in EOC.

### 3.2. The Constitutive Expression of TLR4/MyD88/NF-*κ*Bp65/HIF-1*α* Signals in Human EOC Cell Lines

**Figure 2:**
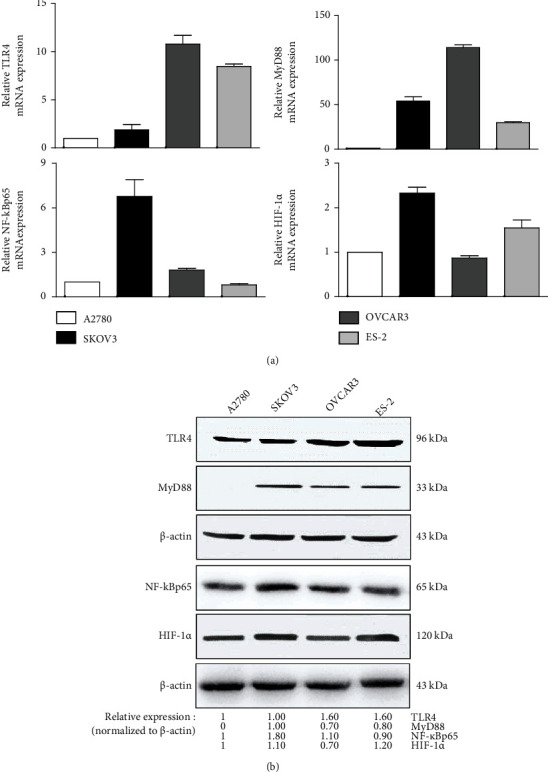
Expression levels of TLR4/MyD88/NF-*κ*Bp65/HIF-1*α* signaling molecules in human EOC cell lines. The (a) mRNA and (b) protein expressions of TLR4, MyD88, NF-*κ*Bp65, and HIF-1*α* in A2780, SKOV3, OVCAR3, and ES-2 cells measured by qPCR assay and western blotting, respectively. Three independent experiments were performed. The representative means and representative images from 3 independent experiments are shown, respectively.

MyD88, the critical adaptor protein, contributed to the development and immune escape of various tumors. Considering the active role of MyD88, the TLR4/NF-*κ*Bp65 signaling pathway can be divided into MyD88-dependent or MyD88-independent pathways. To investigate the exact role of the TLR4/MyD88/NF-*κ*Bp65/HIF-1*α* pathway in the progression of EOC, we first examined the respective constitutive mRNA and protein levels of TLR4, MyD88, NF-*κ*Bp65, and HIF-1*α* in A2780, SKOV3, OVCAR3, and ES-2 cell lines. As shown in Figure 2(b), the broad expressions of the abovementioned proteins were recorded in the 4 EOC cell lines, except for MyD88 in the A2780 cells. No MyD88 expression could be detected even after repeat experimentations. Notable, as compared with the A2780 cells, the SKOV3 cells showed extraordinarily high levels of MyD88, NF-*κ*Bp65, and HIF-1*α*. To elucidate whether the MyD88-dependent and MyD88-independent TLR4/NF-*κ*Bp65 signaling pathways had similar effects on the HIF-1*α* activity of EOC, we used the A2780 and SKOV3 cells as the study cell models for the subsequent analysis.

### 3.3. Upregulating Effects of LPS on HIF-1*α* Activity in Human EOC Cells Were Induced through the TLR4/NF-*κ*B Pathway

The close association between hypoxia and inflammation is already well known. In the present study, we verified whether the TLR4/NF-*κ*Bp65 pathway is involved in mediating the upregulating effects of LPS on the expression of HIF-1*α* in EOC cells.

**Figure 3:**
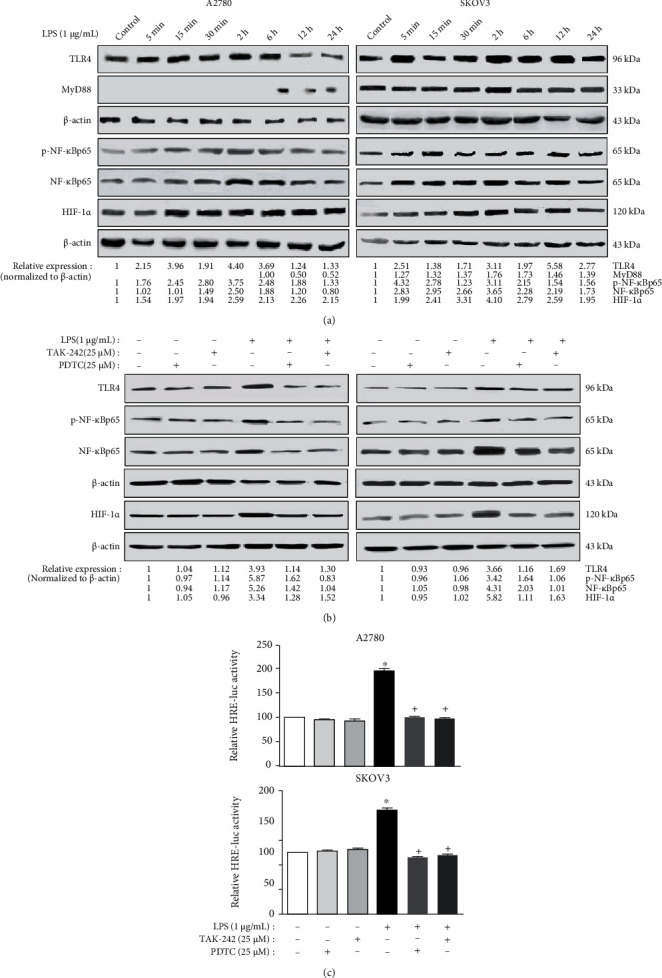
LPS treatment could upregulate the HIF-1*α* expression and activity in human EOC cell lines via TLR4/NF-*κB* signaling. (a) LPS treatment enhanced the levels of HIF-1*α* and TLR4 signaling-related proteins in the human EOC cell lines. After incubation with 1 *μ*g/mL LPS for different designated time spots (5 min, 15 min, 30 min, 2 h, 6 h, 12 h, and 24 h), protein lysates from A2780 and SKOV3 cells were prepared and analyzed by western blotting test. (b) Upregulating effects by LPS on the expression of TLR4, p-NF-*κ*B, NF-*κ*B, and HIF-1*α* could be blocked by TAK-242 or PDTC. The A2780 and SKOV3 cells were pretreated with TAK-242 (TLR4 inhibitor, 25 *μ*M) or PDTC (NF-*κ*B inhibitor, 25 *μ*M) for 1 h, respectively, followed by 1 *μ*g/mL LPS treatment for 6 h. Protein lysates from the cells were prepared and assayed by western blotting. (c) The luciferase reporter test was conducted to assess the HIF-1*α* transcriptional activity. The *HRE* construct and *β*-gal vectors were cotransfected into the cells. After pretreatment with TAK-242 or PDTC for 1 h, cells were then treated with LPS (1 *μ*g/mL) for 6 h. The experiments shown are representative of three independent experiments with similar results. The data shown as the mean ± SD represent the average luciferase activity of 3 wells from one experiment. ^∗^*P* < 0.05 vs. the control group. ^+^*P* < 0.05 vs. the LPS group.

First, LPS, the inflammation inducer and the proven natural ligand of TLRs, was used as an activator of the TLR4/NF-*κ*B pathway in the subsequent analysis. As shown in Figure 3(a), a time-effect study by western blotting analysis showed that the HIF-1*α* level along with the levels of TLR4, NF-*κ*Bp65, and p-NF-*κ*Bp65 in the A2780 and SKOV3 cells was significantly enhanced by treatment with 1 *μ*g/mL LPS for 30 min, 2 h, or 6 h, except for the p-NF-*κ*Bp65 levels in SKOV3 cells (for 5 or 15 min). Notably, the MyD88 expression in A2780 cells could not be detected either with or without LPS treatment for less than 2 h but could be induced by LPS treatment exceeding 6 h. Meanwhile, the level of MyD88 in the SKOV3 cells was slightly enhanced after LPS treatment for 2 h and 6 h. These results indicate that in EOC cells, LPS may activate the MyD88-dependent or MyD88-independent TLR4/NF-*κ*B signaling pathways and induce the expression of HIF-1*α*. For exploring the exact mechanisms underlying these events, the treatment time of LPS was fixed to 6 h in the subsequent analysis.

Next, the TLR4 inhibitor TAK-242 and the NF-*κ*B inhibitor PDTC were used to determine whether the upregulating effects of LPS on the HIF-1*α* level in EOC cells were mediated via TLR4/NF-*κ*B signaling. As illustrated in Figure 3(b), the levels of TLR4, NF-*κ*Bp65, p-NF-*κ*Bp65, and HIF-1*α* in A2780 and SKOV3 cells treated with LPS were obviously greater than those in the control cells. Pretreatment with 25 *μ*M TAK-242 (TLR4 inhibitor) or 25 *μ*M PDTC (NF-*κ*B inhibitor) for 1 h followed by LPS treatment could remarkably block the abovementioned effects, indicating that the TLR4/NF-*κ*B signaling pathway may be involved in LPS-induced HIF-1*α* expression. Third, a luciferase assay was conducted to observe the HIF-1*α* transcriptional activity. Our results suggested that the HIF-1*α* transcriptional activity (indicated by the relative HRE-luc activity in Figure 3(c)) after treatment of LPS was significantly higher than the control group for A2780 and SKOV3 cells. While pretreatment with TAK-242 or PDTC could remarkably block the upregulation effects of LPS. Therefore, our cumulative findings suggest that LPS may promote the HIF-1*α* activity in EOC via the TLR4/NF-*κ*B signaling pathway.

### 3.4. LPS and Hypoxia Stimuli Possess the Synergistic Effects on HIF-1*α* Activity in Human EOC Cells

Accumulating evidence has demonstrated that chronic inflammation and hypoxia stimuli are the two key factors involved in tumor development [4, 23, 24]. After confirming the upregulating effects of LPS on the HIF-1*α* activity in EOC cells, we next investigated the presence of synergetic effects of LPS and hypoxia stimuli.

**Figure 4:**
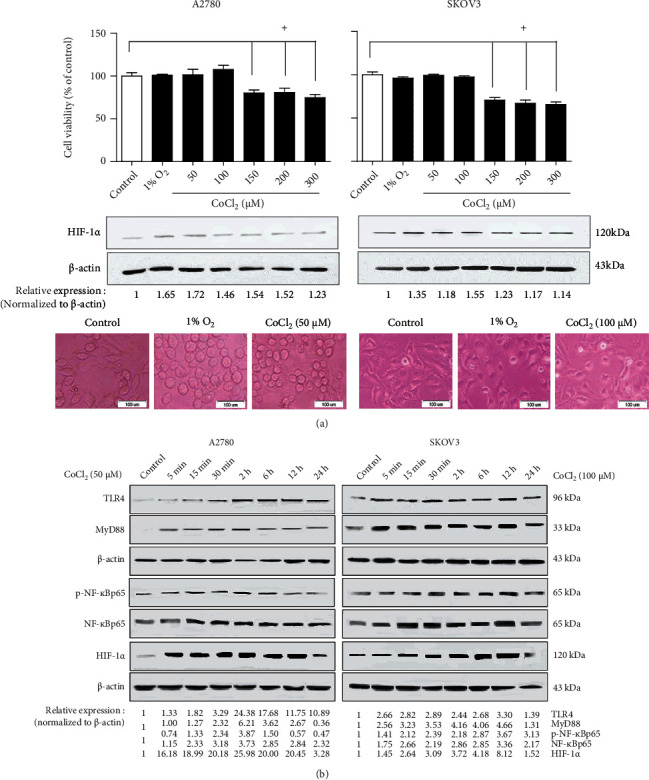
Hypoxia stress upregulates the HIF-1*α* and TLR4 signaling-related protein levels in human EOC cell lines. (a) The mimic effects of CoCl_2_ similar to that of 1% O_2_ occurred at the 50 *μ*M dose for A2780 and at the 100 *μ*M dose for SKOV3, respectively. The human EOC cell lines A2780 and SKOV3 were treated with 1% O_2_ or with different CoCl_2_ doses (50, 100, 150, 200, and 300 *μ*M). After 24 h, cell viability and HIF-1*α* protein level were analyzed by MTT (top panel) and western blotting (middle panel). The cells' morphology under hypoxia was observed by microscopy (bottom panel, magnification ×200). Data are shown as mean ± SD. ^+^*P* < 0.05 vs. the control group. (b) The time-dependent effects of CoCl_2_ exposure on the TLR4, MyD88, HIF-1*α*, p-NF-*κ*B, and NF-*κ*B protein levels in A2780 and SKOV3 are shown. The cells were treated with 50 *μ*M CoCl_2_ (in A2780 cells) or 100 *μ*M CoCl_2_ (in SKOV3 cells) for different designated time slots (5 min, 15 min, 30 min, 2 h, 6 h, 12 h, and 24 h). After treatment, western blotting was performed to assay the protein levels. The representative results from 3 independent experiments are shown.

For this purpose, we first determined the mimic dose-effect and time-effect of CoCl_2_ similar to that of 1% O_2_ by the MTT and western blotting methods. In Figure 4(a), low-dose CoCl_2_ treatment (50 and 100 *μ*M) for 24 h had no effect on EOC cell proliferation, similar to that of 1% O_2_. The HIF-1*α* level in EOC cells increased by different intensities after CoCl_2_ treatment for 24 h (50, 100, 150, 200, and 300 *μ*M), and the maximum effect of CoCl_2_ occurred at the 50 *μ*M dose for A2780 cells and 100 *μ*M dose for SKOV3 cells. Consequently, the working concentrations of 50 *μ*M and 100 *μ*M CoCl_2_ were, respectively, used for A2780 and SKOV3 in the subsequent experiments. Meanwhile, a time-effect study by western blotting showed that the HIF-1*α* expression and the TLR4, MyD88, NF-*κ*Bp65, and p-NF-*κ*Bp65 levels were significantly enhanced by CoCl_2_ treatment for different designated durations. As shown in Figure 4(b), the obvious upregulating effects of CoCl_2_ accrued mainly at the treatment time of 2 h, 6 h, and 12 h. Therefore, we used the LPS treatment time of 6 h (Figure 3(a)) as the CoCl_2_ treating time in the subsequent tests to investigate the synergetic effect of LPS and hypoxia stimuli on EOC cells.

**Figure 5:**
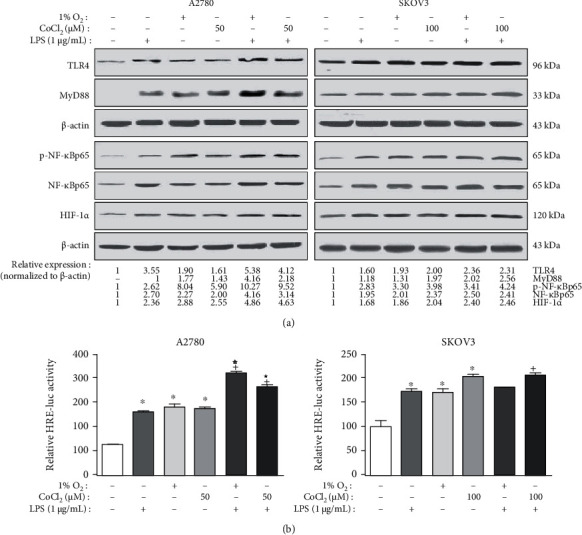
Hypoxia stress could enhance the upregulating effects of LPS on the HIF-1*α* activity in human EOC cells via TLR4/NF-*κ*B signaling. The A2780 and SKOV3 cells were treated without or with 1 *μ*g/mL LPS for 6 h under normoxic (21% O_2_) or hypoxic (1% O_2_ or CoCl_2_) conditions, respectively. After the treatment, western blotting and luciferase reporter assays were, respectively, used to assess the (a) protein levels and the (b) HIF-1*α* transcriptional activity. For (b) the *HRE* construct and *β*-gal vectors were cotransfected into the A2780 and SKOV3 cells. After treatment without or with LPS for 6 h under normoxic (21% O_2_) or hypoxic (1% O_2_ or CoCl_2_) conditions, the values of *β*-gal and luciferase were measured. Data are shown as mean ± SD. The experiments shown are representative of three independent experiments with similar results. The data represent the average luciferase activity of 3 wells from one experiment. ^∗^*P* < 0.05 vs. the control group. ^+^*P* < 0.05 vs. the LPS group. ^☆^*P* < 0.05 vs. the 1% O_2_ group. ^★^*P* < 0.05 vs. the CoCl_2_ group.

Past studies have shown that hypoxia could trigger inflammation and even further aggravate the inflammatory response [18, 25]. Our results confirmed that both 1% O_2_ and CoCl_2_ treatments could enhance the upregulating influence of LPS on the HIF-1*α* activity in human EOC cells via the TLR4/MyD88/NF-*κ*B pathway. As compared to the control group, 1% O_2_ alone, CoCl_2_ alone, or LPS alone treatment could increase the HIF-1*α* expression and the TLR4/MyD88/NF-*κ*Bp65 signaling. Meanwhile, treatment with 1% O_2_ or CoCl_2_ together with LPS, in comparison with treatment with LPS alone, could significantly increase the effects (Figure 5(a)). We further detected the transcriptional activity of HIF-1*α* after LPS or (and) hypoxia treatment. In Figure 5(b), compared with the control groups, hypoxia (1% O_2_ or CoCl_2_) or LPS alone could obviously increase the HRE luciferase activity in both A2780 and SKOV3 cells. Furthermore, treatment with LPS combined with 1% O_2_ or CoCl_2_ could significantly upregulate the transcriptional activity of HIF-1*α* in comparison with LPS alone in A2780 cells. A similar increasing trend was recorded for SKOV3 cells, albeit there was no statistical significance between the treatments with 1% O_2_ together with LPS and with LPS alone. These results indicate that LPS, combined with hypoxia could synergistically enhance the TLR4/MyD88/NF-*κ*Bp65 signaling and the expression and activity of HIF-1*α*.

### 3.5. LPS and Hypoxia Stimuli Induced the Formation of TLR4/NF-*κ*B/HIF-1*α* Signaling Loop in Human EOC Cell Lines

**Figure 6:**
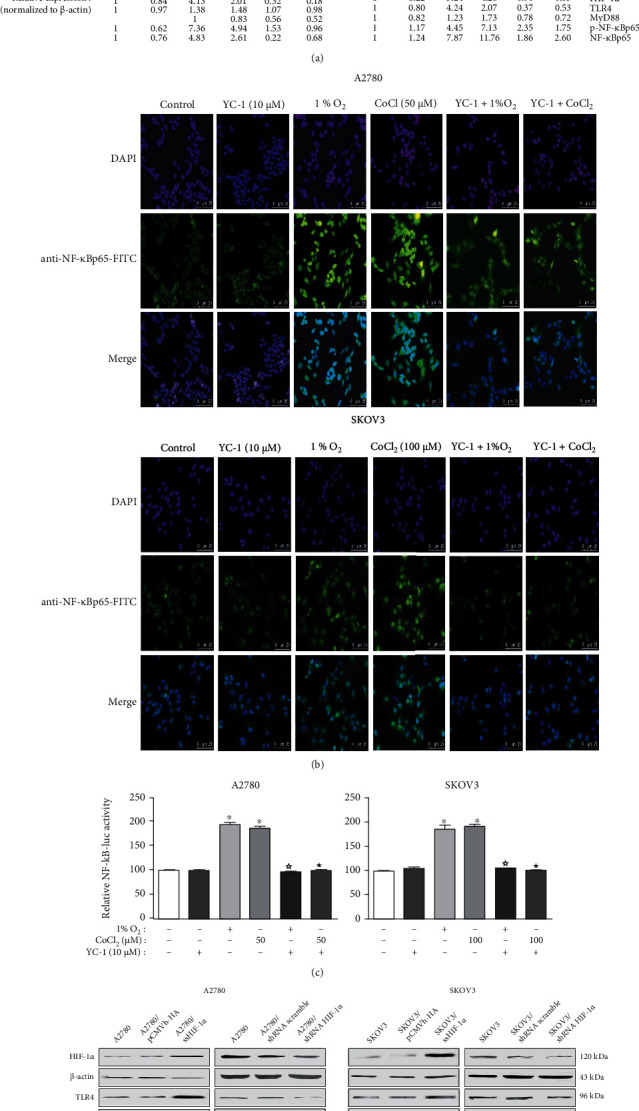
Hypoxic stress upregulates the TLR4/NF-*κ*B signaling in human EOC cell lines via the HIF-1*α* activity. (a) Hypoxic stress-induced TLR4/NF-*κ*B signaling activation can be blocked by YC-1. After treatment with YC-1 (HIF-1*α* inhibitor, 10 *μ*M) for 1 h, A2780 and SKOV3 cells were exposed to normoxia (21% O_2_) or hypoxia (1% O_2_ or CoCl_2_) for 6 h. After the treatment, western blotting was performed to analyze. (b, c) The nuclear translocation and transcriptional activity of NF-*κ*B induced by hypoxia stimulation can be blocked by YC-1. After pretreatment with 10 *μ*M YC-1 for 1 h, the A2780 and SKOV3 cells were exposed to normoxia (21% O_2_) or hypoxia (1% O_2_ or CoCl_2_) conditions for 6 h. (b) Then cells were fixed, incubated with FITC-conjugated anti-NF-*κ*Bp65 to show the localization of NF-*κ*B in cells (green), and counterstained the nuclei with DAPI (blue). (c) The NF-*κ*B construct and *β*-gal vectors were cotransfected into A2780 and SKOV3. The values of *β*-gal and luciferase were detected, and the luciferase activities were normalized to *β*-gal values. The experiments shown are representative of three independent experiments with similar results. The data shown as the mean ± SD represent the average luciferase activity of 3 wells from one experiment. (d) The overexpression or knockdown of HIF-1*α* in the A2780 and SKOV3 caused the enhancement or attenuation of the TLR4/NF-*κ*B signaling, respectively. The A2780 and SKOV3 cells were transiently transfected with pCMVh-HA-ssHIF-1*α* (named the sense HIF-1*α* vector) and empty vector pCMVh-HA or shRNA-HIF-1*α* (named small hairpin RNA targeting HIF-1*α*) and control shRNA, respectively. The protein lysates from the transfectants were prepared and assessed by western blotting. The representative images are shown from 3 independent experiments. ^∗^*P* < 0.05 vs. the control group. ^☆^*P* < 0.05 vs. the 1% O_2_ group. ^★^*P* < 0.05 vs. the CoCl_2_ group.

HIF-1*α* plays a vital part in the initial and developing stages of tumor metastasis. As confirmed by other researchers and based on our results, both hypoxia and LPS stimuli contribute to the activation of HIF-1*α* via the NF-*κ*B signaling pathway [26–28]. We therefore investigated whether hypoxia could regulate the activation of TLR4/NF-*κ*B signaling via HIF-1*α*. First, pretreatment with YC-1 (10 *μ*M), the commonly used specific blocker of HIF-1*α*, for 1 h could remarkably block the upregulating effects of 1% O_2_ or CoCl_2_ on the TLR4/NF-*κ*B signals with the coconcurrent blockage of HIF-1*α*, indicating that hypoxia could indirectly affect the TLR4/NF-*κ*B signals via HIF-1*α* (Figure 6(a)). Moreover, pretreatment with YC-1 for 1 h could remarkably block the nucleus accumulation and transcriptional activity of NF-*κ*B induced by 1% O_2_ or CoCl_2_, respectively (Figures 6(b) and 6(c)). Second, the sense HIF-1*α* vector (pCMVh-HA-ssHIF-1*α*), small hairpin RNA targeting HIF-1*α* (shRNA-HIF-1*α*) vector, and the corresponding empty vectors were, respectively, used to overexpress or knock down the HIF-1*α* expression in A2780 and SKOV3 endogenously. As illustrated in Figure 6(d), the overexpression or knockdown of HIF-1*α* led to the enhancement or attenuation of the TLR4/NF-*κ*B signaling in A2780 and SKOV3 cells, respectively. Based on the abovementioned results (Figures 3, 5, and 6), LPS induced HIF-1*α* expression and activity via the TLR4/NF-*κ*B signaling pathway. Meanwhile, HIF-1*α*, a key transcriptional factor to hypoxia, could activate the TLR4/NF-*κ*B signaling pathway by upregulating the expression of TLR4. Therefore, we speculated that the LPS and hypoxia stimuli induced the formation of TLR4/NF-*κ*B/HIF-1*α* positive feedback loop and that this signaling loop may exist and contribute to the EOC development.

### 3.6. In Vivo Study of the TLR4/NF-*κ*B/HIF-1*α* Signaling Loop Induced by LPS

**Figure 7:**
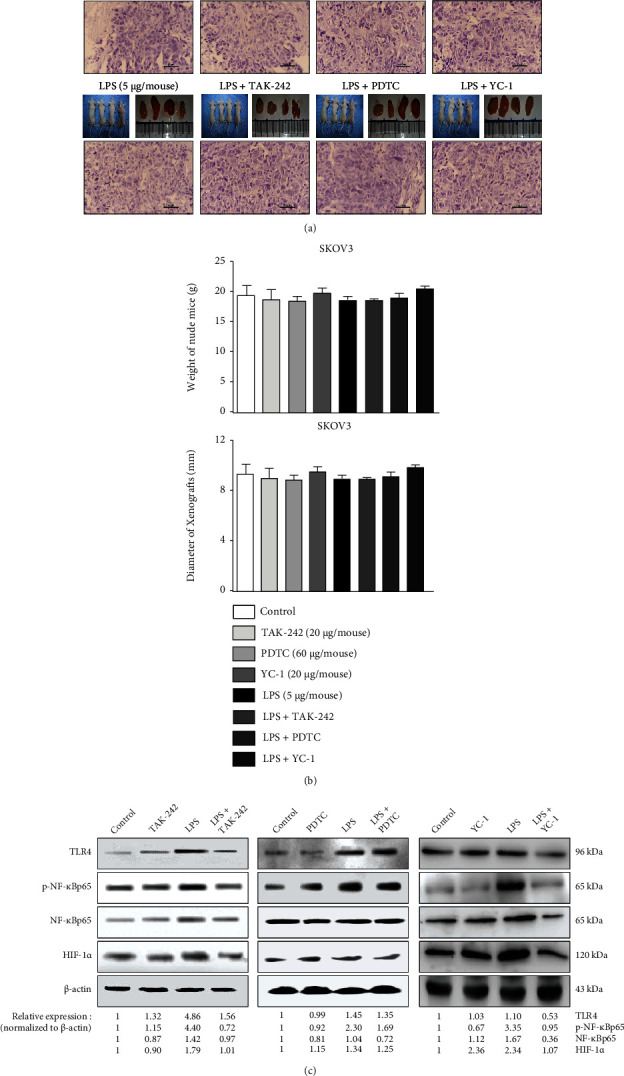
The upregulating effects of LPS on the TLR4/NF-*κB*/HIF-1*α* signaling loop exists in the murine EOC model. The SKOV3 cells were prepared for injection into the right-sided flanks of the nude mice. After 21 days, when the EOC tumors grew to a diameter of approximately 9 mm, TAK-242 (TLR4 inhibitor, 20 *μ*g/mouse), PDTC (NF-*κ*B inhibitor, 60 *μ*g/mouse), or YC-1 (HIF-1*α* inhibitor, 20 *μ*g/mouse) were injected into the subcutaneous tumor basement, respectively. After 1 h, LPS (5 *μ*g/mouse) was injected by the same method, and 24 h later, the mice were sacrificed. (a) Tumor nodes obtained by surgery (top panel) and the sections prepared for HE staining (bottom panel). (b) Statistical analysis on the weight of tumor-bearing mice and the diameter of tumors. (c) The protein lysates were extracted from the tumor tissues, and then western blotting was performed to assay the levels of TLR4, p-NF-*κ*Bp65, NF-*κ*B, and HIF-1*α*. Three independent experiments were performed, and the representative images are shown. Data represents the mean ± SD from 3 independent experiments.

To explore the impact of LPS on the TLR4/NF-*κ*B/HIF-1*α* signaling loop of EOC in vivo, SKOV3 cell suspension was injected into the flanks of nude mice. After 3 weeks of injecting, TAK-242 (TLR4 inhibitor, 20 *μ*g/mouse), PDTC (NF-*κ*B inhibitor, 60 *μ*g/mouse), or YC-1 (HIF-1*α* inhibitor, 20 *μ*g/mouse) were injected into the subcutaneous tumor basement, and, after an hour, LPS (5 *μ*g/mouse) was injected by the same method. After 24 h, the mice were sacrificed to evaluate the expression levels of the TLR4/NF-*κ*B/HIF-1*α* in tumor tissues. As illustrated in Figure 7(a), the morphological results of solid EOC tumors and HE staining suggested the successful construction of the murine EOC model. The tumor tissue taken from the nude mice showed obvious malignant tumor atypia, which is the same as the primary ovary cancer. Statistical analysis on the weight of tumor-bearing mice and the diameter of tumors showed no difference (Figure 7(b)) among the groups. Notably, western blotting analysis was conducted on protein extraction from tumor samples and revealed significant blockage caused by TAK-242, PDTC, and YC-1 on the TLR4/NF-*κ*B/HIF-1*α* signaling loop (Figure 7(c)).

## 4. Discussion

TLR4, which recognizes LPS, initiates the innate immune and inflammatory responses. The transcription factor, HIF-1*α*, plays a key role in hypoxia-related disease. It is well known that TLR4 and HIF-1*α* contribute to the progression of numerous inflammatory diseases, including cancer [18, 20]. In this study, we revealed that TLR4 could induce the expression and activation of HIF-1*α* via NF-*κ*B and that HIF-1*α* could also directly regulate the TLR4/NF-*κ*B pathway. The cross talk between TLR4/NF-*κ*B and HIF-1*α* signaling pathway forms a positive feedback loop and contributes to tumor initiation, malignant progression, and prognosis in EOC.

In recent years, enhanced TLR4 expression was identified in several tumors, including pancreatic cancer, colorectal cancer, and esophageal cancer, among others [29–31]. Moreover, the TLR4 expression was correlated with increased levels of NF-*κ*B and HIF-1*α*, higher clinical staging, and dramatically reduced patient survival [20]. Kelly et al. [7] first reported that the expression of TLR4 in EOC cells and the chemoresistance to paclitaxel are mediated by the TLR4/MyD88 signaling. Furthermore, the level of TLR4 was positively associated with the key markers of the NF-*κ*B signaling pathway, while the coexpression of TLR4/MyD88/NF-*κ*B was correlated with poor prognosis in EOC patients [32, 33]. A recent investigation demonstrated that the HIF-1*α* level is markedly enhanced in ovarian cancer in comparison with that in normal ovarian tissues and that the enhanced expression of HIF-1*α* is related to markedly shorter overall patient survival. Furthermore, the expression level of HIF-1*α* is associated with chemoresistance in a metastatic and aggressive ovarian tumor. Thus, the high level of HIF-1*α* may be taken as a prognostic marker in ovarian cancer [34, 35]. However, the potential associations with TLR4/NF-*κ*B and HIF-1*α* remain largely unknown in EOC. In this study, we noted elevated expressions of TLR4, NF-*κ*Bp65, and HIF-1*α* in EOC specimens, and their expressions were significantly correlated with the histological differentiation degree of the tumor. We further demonstrated a positive correlation among TLR4, NF-*κ*Bp65, and HIF-1*α*, indicating a possible cross talk between TLR4 signaling and the HIF-1 pathway, which acted synergistically to promote the progression of EOC.

MyD88, a key adaptor molecule, plays critical roles in TLR4 signaling. The expression of MyD88 was detected in approximately 70% of EOC patients, and it has been considered a significantly poor prognostic indicator of tumor [36, 37]. In this study, we selected the MyD88(-) A2780 cells and MyD88(+) SKOV3 cells for further analysis. Our results indicated that LPS could activate the TLR4/NF-*κ*B signaling pathway in both A2780 and SKOV3 cells. Both MyD88-dependent and MyD88-independent pathways are known to mediate the TLR4/NF-*κ*B signaling pathway following LPS response. Although TLR4 signaling through MyD88 activates the early phase of NF-*κ*B, the MyD88-independent pathway also triggers the TLR4-mediated late-phase activation of NF-*κ*B [9]. This finding is consistent with our observation of earlier NF-*κ*B P65 phosphorylate peaks in SKOV3 cells than in the A2780 cells. Unexpectedly, our results showed that LPS could induce the MyD88 protein expression in MyD88(-) A2780 cells by exceeding 6 h of stimulation. It has been demonstrated that MyD88, an essential signaling component of the TLR4 pathway, mediates paclitaxel (PTX) resistance [7]. PTX signaling via TLR4/MyD88 induced the NF-*κ*B pathway activation, which has been linked to the upregulation of antiapoptotic protein expression and an increase in cellular proliferation [8]. In addition, MyD88-positive EOC cells have a functioning TLR4/MyD88 pathway and are possibly indicative of an ovarian cancer stem cell that is highly resistant to proapoptotic signaling [38]. Accordingly, we proposed that inflammation may be involved in the acquisition of chemoresistance through the MyD88 expression in ovarian cancer cells. However, further research is warranted to confirm this speculation. Meanwhile, our results indicated that in both A2780 and SKOV3 cells, the expression levels of TLR4 decreased 24 h after LPS treatment. It is possible that the endocytosis of the receptor following the ligand-receptor interaction results in lysosomal degradation, which leads to the controlled duration of TLR4 signaling [39] In addition, ubiquitinated degradation is related to the downregulation of TLR4. Triad3A, an E3 ubiquitin-protein ligase, was found to bind the cytoplasmic domain of TLR4 to promote the ubiquitylation and degradation of TLR4 [40]. We further revealed HIF-1*α* expression and activation in ovarian cancer cells following LPS stimulation, which also involves the TLR-induced NF-*κ*B activation. Previous studies have indicated that LPS-induced HIF-1*α* stabilization is mediated by the TLR4/NF-*κ*B signaling pathway under normoxia in immune cells and oral squamous cell carcinoma [21, 27, 28]. Indeed, NF-*κ*B could directly bind with the promoter of HIF-1*α* located at position 197/188 upstream of the transcription start site and modulate the expression of HIF-1*α*. Mutation of this site resulted in a significant reduction in response to p65, confirming that this site is functional [41, 42]. Within the present study, when TLR4 or NF-*κ*B was inhibited pharmacologically, our results showed that the protein levels and transcriptional activity of HIF-1*α* were downregulated after LPS-induction. Therefore, the present study suggests for the first time the existence of the TLR4-NF-*κ*B-HIF-1*α* signaling pathway in ovarian cancer cells, which implicates an important association between the inflammatory pathway and hypoxia-response pathway.

It is well known that hypoxia stabilizes the inducible subunit HIF-1*α*, which is rapidly degraded via the ubiquitin-proteasome pathway under normoxia. Interestingly, short-term hypoxia increases the HIF-1*α* levels not only by stabilizing HIF-1*α* protein but also by inducing HIF-1*α* gene transcription in the pulmonary artery smooth muscle cells. This mechanism involves the activation of NF-*κ*B and its binding to the HIF-1*α* promoter located at position 197/188 [26]. In contrast, as per a diverse range of studies, the predominant mode of HIF-1 regulation occurs at the level of protein stability. Hypoxia has no influence on the levels of HIF-1*α* mRNA. The reason for obtaining the different results on HIF-1*α* mRNA is related to the rapid but transient increase of HIF-1*α* mRNA by hypoxia. Given the transient nature of this response, the inappropriate exposure time may have prevented the detection of HIF-1 mRNA upregulation [26]. Another explanation for the controversial data was proposed that a cis-repressor acting as an antagonist on an upregulating element within the HIF-1 promoter may prevent transcription of the HIF-1 gene in a cell type-specific way [43]. In addition, the NF-*κ*B basal activity is a prerequisite to accumulating HIF-1*α* protein under hypoxia in cultured cells or tissue. The complete block of the NF-*κ*B activation pathway prevented hypoxia-induced HIF-1*α* [44, 45]. Therefore, NF-*κ*B may be essential for the extent and speed of HIF-1*α* activation following hypoxic insult. In accordance with our results, CoCl_2_ treatment (a hypoxia mimetic) activated NF-*κ*B and induced HIF-1*α* protein expression in both A2780 and SKOV3 ovarian cancer cells. As per these results, we considered that hypoxia could activate the NF-*κ*B signaling pathway and contribute to the regulation of inflammation in EOC.

In addition to activating NF-*κ*B, hypoxia could enhance the response of susceptibility to inflammatory signals by upregulating TLR4. In macrophages, HIF-1*α* transcriptionally regulates the TLR4 level under hypoxia via binding to the TLR4 promoter. The mouse TLR4 promoter contains a hypoxia-responsive element (HRE) located at position -407 to -404, while the human TLR4 promoter containing at least two HREs is located at the positions -2811 to -2814 and -1185 to -1188. Therefore, hypoxia augmented the cell responsiveness to bacterial components owing to the increased TLR4 expression in macrophages [18]. In addition, further investigation demonstrated that PI3K/Akt at least partially contributes to hypoxia-induced TLR4 expression via regulating HIF-1*α* accumulation and transcriptional activation [19]. Our results also showed that the expression levels of TLR4 were augmented on exposure to hypoxic stress. Moreover, hypoxia treatment augmented the responsiveness of ovarian cancer cells to LPS, including the TLR4 expression level and the downstream NF-*κ*B and HIF-1*α* activities. Here, we further demonstrated that the hypoxia-mediated TLR4 expression and downstream NF-*κ*B transcriptional activity were HIF-1*α*-dependent by blocking the HIF-1*α* accumulation with YC-1. Meanwhile, the ability of the HIF-1*α* expression plasmid to enhance the TLR4 expression and the ability of HIF-1*α* shRNA to inhibit the TLR4 expression provided additional evidence to sustain the role of HIF-1*α* on regulating the expression of TLR4 in ovarian cancer cells. In accordance with our results, recent evidences have shown that HIF-1*α* could mediate the expression of TLR4 in pancreatic cancer, oral squamous cell carcinoma, and hepatocellular carcinoma under hypoxia. These studies revealed the critical effect of the HIF-1*α*-mediated TLR4-NF-*κ*B signaling pathway in tumor growth and progression [21, 46, 47].

## 5. Conclusions

In the present study, we noted a strong positive correlation among TLR4, NF-*κ*Bp65, and HIF-1*α* in EOC specimens, indicating the existence of a cross talk between the TLR4/NF-*κ*B and HIF-1*α* signaling pathways. Our in vitro results showed that the TLR4 signaling pathway induced the HIF-1*α* protein expression and enhanced the HIF-1*α* transcription activity via NF-*κ*B. Moreover, HIF-1*α* could activate the TLR4/NF-*κ*B signaling pathway by upregulating the expression of TLR4. The in vivo transplantation model confirmed that TLR4 and HIF-1*α* mutually regulated each other in EOC. Combined with the results, we believe that TLR4-mediated HIF-1*α* activation and HIF-1*α*-induced TLR4 expression form a positive feedback loop that is closely associated with inflammation and hypoxia, which in turn can synergistically promote the development of EOC. Our findings may provide more promising therapeutic strategies for epithelial ovarian cancer based on the inhibition of the TLR4/NF-*κ*B/HIF-1 loop.

## Data Availability

All data in this published article are available from the corresponding author on reasonable request.

## References

[1] Vaughan S., Coward J. I., Bast R. C. (2011). Rethinking ovarian cancer: recommendations for improving outcomes. *Nature Reviews. Cancer*.

[2] Giampaolino P., Della L. C., Foreste V. (2019). Unraveling a difficult diagnosis: the tricks for early recognition of ovarian cancer. *Minerva Medica*.

[3] Luo N., Guo J., Chen L., Yang W., Qu X., Cheng Z. (2016). ARHGAP10, downregulated in ovarian cancer, suppresses tumorigenicity of ovarian cancer cells. *Cell Death & Disease*.

[4] Finger E. C., Giaccia A. J. (2010). Hypoxia, inflammation, and the tumor microenvironment in metastatic disease. *Cancer and Metastasis Reviews*.

[5] Sato Y., Goto Y., Narita N., Hoon D. S. (2009). Cancer cells expressing toll-like receptors and the tumor microenvironment. *Cancer Microenvironment*.

[6] Yu L., Chen S. (2008). Toll-like receptors expressed in tumor cells: targets for therapy. *Cancer Immunology, Immunotherapy*.

[7] Kelly M. G., Alvero A. B., Chen R. (2006). TLR-4 signaling promotes tumor growth and paclitaxel chemoresistance in ovarian cancer. *Cancer Research*.

[8] Szajnik M., Szczepanski M. J., Czystowska M. (2009). TLR4 signaling induced by lipopolysaccharide or paclitaxel regulates tumor survival and chemoresistance in ovarian cancer. *Oncogene*.

[9] Akira S., Takeda K. (2004). Toll-like receptor signalling. *Nature Reviews. Immunology*.

[10] Karin M. (2008). The I*κ*B kinase - a bridge between inflammation and cancer. *Cell Research*.

[11] Suhail M., Tarique M., Muhammad N. (2021). A critical transcription factor NF-*κ*B as a cancer therapeutic target and its inhibitors as cancer treatment options. *Current Medicinal Chemistry*.

[12] Vaupel P., Mayer A. (2007). Hypoxia in cancer: significance and impact on clinical outcome. *Cancer Metastasis Reviews*.

[13] Semenza G. L. (2010). Defining the role of hypoxia-inducible factor 1 in cancer biology and therapeutics. *Oncogene*.

[14] Blouin C. C., Pagé E. L., Soucy G. M., Richard D. E. (2004). Hypoxic gene activation by lipopolysaccharide in macrophages: implication of hypoxia-inducible factor 1*α*. *Blood*.

[15] Spirig R., Djafarzadeh S., Regueira T. (2010). Effects of TLR agonists on the hypoxia-regulated transcription factor HIF-1*α* and dendritic cell maturation under normoxic conditions. *PLoS One*.

[16] Riddell J. R., Maier P., Sass S. N., Moser M. T., Foster B. A., Gollnick S. O. (2012). Peroxiredoxin 1 stimulates endothelial cell expression of VEGF via TLR4 dependent activation of HIF-1*α*. *PLoS One*.

[17] Balamurugan K. (2016). HIF-1 at the crossroads of hypoxia, inflammation, and cancer. *International Journal of Cancer*.

[18] Kim S. Y., Choi Y. J., Joung S. M., Lee B. H., Jung Y. S., Lee J. Y. (2010). Hypoxic stress up-regulates the expression of toll-like receptor 4 in macrophages via hypoxia-inducible factor. *Immunology*.

[19] Kim S. Y., Jeong E., Joung S. M., Lee J. Y. (2012). PI3K/Akt contributes to increased expression of toll-like receptor 4 in macrophages exposed to hypoxic stress. *Biochemical and Biophysical Research Communications*.

[20] Zhang J.-J., Wu H.-S., Wang L., Tian Y., Zhang J.-H., Wu H.-L. (2010). Expression and significance of TLR4 and HIF-1*α* in pancreatic ductal adenocarcinoma. *World journal of gastroenterology: WJG*.

[21] Han S., Xu W., Wang Z. (2016). Crosstalk between the HIF-1 and toll-like receptor/nuclear factor-*κ*B pathways in the oral squamous cell carcinoma microenvironment. *Oncotarget*.

[22] Zhou Z., Zhu X., Chen J., Yang S., Sun R., Yang G. (2014). The interaction between toll-like receptor 4 signaling pathway and hypoxia- inducible factor 1*α* in lung ischemia-reperfusion injury. *The Journal of Surgical Research*.

[23] Coussens L. M., Werb Z. (2002). Inflammation and cancer. *Nature*.

[24] Chan D. A., Giaccia A. J. (2007). Hypoxia, gene expression, and metastasis. *Cancer Metastasis Reviews*.

[25] Tazzyman S., Murdoch C., Yeomans J., Harrison J., Muthana M. (2014). Macrophage-mediated resp onse to hypoxia in disease. *Hypoxia*.

[26] Belaiba R. S., Bonello S., Zahringer C. (2007). Hypoxia up-regulates hypoxia-inducible factor-1alpha transcription by involving phosphatidylinositol 3-kinase and nuclear factor kappaB in pulmonary artery smooth muscle cells. *Molecular Biology of the Cell*.

[27] Jantsch J., Wiese M., Schödel J. (2011). Toll-like receptor activation and hypoxia use distinct signaling pathways to stabilize hypoxia-inducible factor 1*α* (HIF1A) and result in differential HIF1A-dependent gene expression. *Journal of Leukocyte Biology*.

[28] Ramanathan M., Luo W., Csóka B. (2009). Differential regulation of HIF-1*α* isoforms in murine macrophages by TLR4 and adenosine A2A receptor agonists. *Journal of Leukocyte Biology*.

[29] Park H., Lee Y., Oh Y. (2011). Pancreatic adenocarcinoma upregulated factor promotes metastasis by regulating TLR/CXCR4 activation. *Oncogene*.

[30] Wang E., Qian Z.-R., Nakasono M. (2010). High expression of toll-like receptor 4/myeloid differentiation factor 88 signals correlates with poor prognosis in colorectal cancer. *British Journal of Cancer*.

[31] Sheyhidin I., Nabi G., Hasim A. (2011). Overexpression of TLR3, TLR4, TLR7 and TLR9 in esophageal squamous cell carcinoma. *World Journal of Gastroenterology: WJG*.

[32] Kim K. H., Jo M. S., Suh D. S. (2012). Expression and significance of the TLR4/MyD88 signaling pathway in ovarian epithelial cancers. *World Journal of Surgical Oncology*.

[33] Li Z., Block M. S., Vierkant R. A. (2016). The inflammatory microenvironment in epithelial ovarian cancer: a role for TLR4 and MyD88 and related proteins. *Tumor Biology*.

[34] Shen W., Li H., Liu L., Cheng J. (2017). Expression levels of PTEN, HIF-1*α*, and VEGF as prognostic factors in ovarian cancer. *European Review for Medical and Pharmacological Sciences*.

[35] Gomez-Roman N., Sahasrabudhe N. M., McGregor F., Chalmers A. J., Cassidy J., Plumb J. (2016). Hypoxia-inducible factor 1 alpha is required for the tumourigenic and aggressive phenotype associated with Rab25 expression in ovarian cancer. *Oncotarget*.

[36] Zhu Y., Huang J.-M., Zhang G.-N., Zha X., Deng B.-F. (2012). Prognostic significance of MyD88 expression by human epithelial ovarian carcinoma cells. *Journal of Translational Medicine*.

[37] d'Adhemar C. J., Spillane C. D., Gallagher M. F. (2014). The MyD88+ phenotype is an adverse prognostic factor in epithelial ovarian cancer. *PLoS One*.

[38] Alvero A. B., Chen R., Fu H. H. (2009). Molecular phenotyping of human ovarian cancer stem cells unravels the mechanisms for repair and chemoresistance. *Cell Cycle*.

[39] Husebye H., Halaas Ø., Stenmark H. (2006). Endocytic pathways regulate toll-like receptor 4 signaling and link innate and adaptive immunity. *The EMBO Journal*.

[40] Chuang T. H., Ulevitch R. J. (2004). Triad3A, an E3 ubiquitin-protein ligase regulating toll-like receptors. *Nature Immunology*.

[41] Görlach A., Bonello S. (2008). The cross-talk between NF-*κ*B and HIF-1: further evidence for a significant liaison. *The Biochemical Journal*.

[42] Yoshida T., Hashimura M., Mastumoto T. (2013). Transcriptional upregulation of HIF-1*α* by NF-*κ*B/p65 and its associations with *β*-catenin/p300 complexes in endometrial carcinoma cells. *Laboratory Investigation*.

[43] Minet E., Ernest I., Michel G. (1999). HIF1A gene transcription is dependent on a core promoter sequence encompassing activating and inhibiting sequences located upstream from the transcription initiation site and cis elements located within the 5'UTR. *Biochemical and Biophysical Research Communications*.

[44] Rius J., Guma M., Schachtrup C. (2008). NF-*κ*B links innate immunity to the hypoxic response through transcriptional regulation of HIF-1*α*. *Nature*.

[45] Van Uden P., Kenneth N. S., Rocha S. (2008). Regulation of hypoxia-inducible factor-1*α* by NF-*κ*B. *Biochemical Journal*.

[46] Fan P., Zhang J.-J., Wang B. (2012). Hypoxia-inducible factor-1 up-regulates the expression of toll-like receptor 4 in pancreatic cancer cells under hypoxic conditions. *Pancreatology*.

[47] Zhang X., Li S., Li M., Huang H., Li J., Zhou C. (2016). Hypoxia-inducible factor-1*α* mediates the toll-like receptor 4 signaling pathway leading to anti-tumor effects in human hepatocellular carcinoma cells under hypoxic conditions. *Oncology Letters*.

[48] Guo X., Zhao B., Niu X. (2021). *TLR4 agonist and hypoxia synergistically promote the formation of TLR4/NF-κB/HIF-1α loop in human epithelial ovarian cancer*.

